# The correlation between the ultrasound examination parameters and the pathological characteristics of papillary thyroid carcinomas

**DOI:** 10.12669/pjms.41.3.10566

**Published:** 2025-03

**Authors:** Lei Yang, Yang Luo, Zhiyong Li

**Affiliations:** 1 Lei Yang Department of Functional, Chengdu Shuangliu Hospital of Traditional Chinese Medicine, Chengdu, Sichuan Province 610000, P.R. China; 2 Yang Luo Department of Ultrasound, Chengdu First People’s Hospital, Chengdu, Sichuan Province 610000, P.R. China; 3 Zhiyong Li Department of Functional, Chengdu Shuangliu Hospital of Traditional Chinese Medicine, Chengdu, Sichuan Province 610000, P.R. China

**Keywords:** Papillary thyroid carcinomas, Pathological characteristics, Ultrasound examination parameters

## Abstract

**Objective:**

To evaluate the correlation between ultrasound (US) examination parameters and pathological characteristics of papillary thyroid carcinomas (PTC).

**Methods:**

A retrospective analysis was conducted using clinical data from 89 patients with PTC (malignant group) and 89 patients with benign thyroid nodules (benign group) who underwent US at Chengdu Shuangliu Hospital of Traditional Chinese Medicine between February 2021 to February 2023. The correlation between ultrasound parameters and pathological features of PTC was analyzed.

**Results:**

Ultrasound parameters in the malignant group were significantly different from those in the benign group (*P*<0.05). Peak systolic blood flow velocity (PSV), pulsation index (PI), and resistance index (RI) were significantly higher in patients with malignant lymph node metastasis (LNM) and stage III-IV PTC. In contrast, peak intensity (Peak), mean transit time (MTT), time to peak (TTP), and area under curve (AUC) were significantly lower than those in patients without LNM and stage I-II (*P*<0.05). Spearman’s analysis revealed significant correlations between ultrasound parameters, LNM, disease staging, and gene mutations (*P<0.05*). Of the 89 PTC patients, 27 had disease recurrence, and five died during the follow-up. The poor prognosis group had significantly higher PSV, PI, and RI and lower Peak, MTT, TTP, and AUC compared to the good prognosis group (*P*<0.05).

**Conclusions:**

Ultrasound is a valuable tool for diagnosing and evaluating papillary thyroid carcinoma (PTC). It shows strong correlations between ultrasound parameters and pathological features, including lymph node metastasis and disease staging, aiding early diagnosis and prognosis prediction.

## INTRODUCTION

Papillary thyroid carcinoma (PTC) is the most common type of thyroid cancer,^1^,^2^ with its incidence steadily increasing globallyication of pathological features and differentiation between benign and malignant thyroid tumors are critical for guiding clinical management and treatment strategies.^3^,^4^ RTC is often characterized by genetic alterations, including Rearranged during Transfection (RET) oncogene rearrangements, mutations in the BRAF, telomerase reverse transcriptase (TERT), and RAS genes, in thyroid carcinogenesis. These genetic changes lead to numerous PTC variants with distinct pathological features,^5^ and are emerging as significant markers for both diagnosis and prognosis.^6^,^7^

Although pathological examination is a gold standard for diagnosing thyroid tumors, it is invasive, lacks repeatability, and is not ideal for monitoring tumor progression.^8^,^9^ In contrast, ultrasound (US) has become increasingly used in diagnosing and assessing PTC, as it offers a noninvasive, reliable method to evaluate blood flow distribution within and around thyroid nodules, which aids in differentiating between benign and malignant lesions.^10^–^12^ The characteristic US features of PTC include hypoechogenicity, spiculated or microlobulated margins, microcalcifications, and nonparallel orientation. However, certain PTC variants, such as the follicular variant, may present differently in terms of biological behavior and clinical outcomes.^13^ Notably, current American Thyroid Association (ATA) guidelines^14^ do not provide detailed information on the imaging characteristics of PTC variants.

Several studies have examined the diagnostic and prognostic value of US in PTC, but results have been conflicting. While some studies suggest that ultrasonographic features of PTC subtypes can provide valuable insights into tumor biology and help guide individualized management,^15^ others report that Ultrasound features are not effective in distinguishing certain PTC subtypes.^16^ This study aimed to clarify the correlation between Ultrasound examination parameters, pathological characteristics, and genetic profiles of PTC, seeking to enhance the accuracy of non-invasive diagnostic and prognostic tools for this prevalent malignancy.

## METHODS

Clinical data from 89 patients with papillary thyroid carcinoma (designated as a malignant group) and 89 patients with benign thyroid nodules (designated as a benign group) were retrospectively analyzed. These patients underwent ultrasound examination at Chengdu Shuangliu Hospital of Traditional Chinese Medicine from February 2021 to February 2023.

### Ethical Approval:

This study was conducted in compliance with the ethical standards of the institution and/or National Research Council, as well as the Helsinki Declaration (revised in 2013). It was approved by the Medical Ethics Committee of Shuangliu District Traditional Chinese Medicine Hospital in Chengdu (Ethics No.: ZYY202306; Dated: March 15, 2022). Due to the retrospective nature of observation and review, the informed consent form was not required.

### Inclusion criteria:

- Diagnosis of benign thyroid nodules or papillary thyroid carcinoma (PTC) confirmed through clinical and pathological evaluation, according to the 2017 American Joint Committee on Cancer (AJCC/TNM) staging system, the American Thyroid Association (ATA) guidelines, and the ESMO Clinical Practice Guidelines for Thyroid Cancer.^14^
- Complete clinical and imaging data available.
- For PTC patients, genotyping of the thyroid lesion was performed.

### Exclusion criteria:

- Benign and malignant tumors.
- Presence of cardiovascular and cerebrovascular diseases.
- Hepatitis B, pulmonary tuberculosis and other infectious diseases.
- Mental illness.
- Lactation and pregnancy.
- Previous head and neck examinations or radiation therapy; treatments that may affect the observation indicators
- Secondary thyroid tumors.
- Blood system diseases.

### Ultrasound examination:

The equipment selected was Philips EPIQ color Doppler ultrasound diagnostic instrument. The patient was instructed to lie flat and with neck fully exposed. The probe frequency was set between 6-13 MHz. The ultrasound probe was placed on the patient’s neck to assess lymph node condition, measure the length and ratio of lymph nodes, and evaluate calcification and cystic lesions. The blood flow distribution within the lesion was recorded in detail. A large blood vessel from the lesion tissue was selected for Doppler analysis, and a sampling volume was placed parallel to the vessel’s diameter.

The pulse Doppler blood flow spectrum was obtained, and parameters such as Peak systolic blood flow velocity (PSV), Pulsatility Index (PI), and Resistance Index (RI) were calculated. Following the initial assessment, contrast-enhanced ultrasound (CEUS) was performed. Multiple longitudinal, transverse, and oblique sections of the thyroid and surrounding areas were scanned. After the preliminary diagnosis, the mode was switched to contrast-enhanced ultrasound. A 2 ml ultrasound contrast agent was injected through the superficial vein of the elbow via syringe, followed by a 5 ml flush of physiological saline. The patient’s head and the ultrasound probe were kept stable during the examination, with continuous observation for ≥ 3 minutes. The area of interest was selected, and the time-intensity curve was obtained. Quantitative analysis of ultrasound contrast parameters was performed, including Peak intensity (Peak), Mean transit time (MTT), Time to peak (TTP), and Area under curve (AUC). Notably, AUC was used as a parameter for contrast-enhanced ultrasound (CEUS), not as a conventional ultrasound parameter.

Lymph node metastasis (LNM) was assessed based on ultrasound characteristics such as abnormal lymph nodes with irregular shape, loss of the normal cortex-medulla boundary, or calcifications. The presence of LNM was confirmed by the observed features on ultrasound imaging. The disease staging was based on the 2017 American Joint Committee on Cancer (AJCC/TNM) staging system,^17^ which classifies the disease into stages I-IV based on tumor size, lymph node involvement, and the presence of distant metastasis.

For PTC patients, genetic profiling of the thyroid lesions was conducted by extracting DNA from tissue samples obtained during surgery. The BRAF, RAS, TERT, and RET/PTC mutations were assessed using targeted sequencing techniques. These mutations were analyzed for their correlation with ultrasound parameters, disease stage, and prognosis.

### Collected data:

- Baseline data, including gender, age, tumor diameter, and body mass index (BMI).
- Ultrasound examination parameters, including PSV, PI, RI, Peak, MTT, TTP, and AUC.
- Different pathological features, including malignant LNM, disease staging, and gene mutations.
- Prognostic situation. After a follow-up of six months, the disease recurrence and the deceased were classified as having a poor prognosis. The rest of the cases were classified as having a good prognosis.

### Statistical Analysis:

Data analysis was performed using SPSS version 21.0 software (IBM, Armonk, New York, USA). Measurement data were presented as mean ± standard deviation (SD) and compared between two independent samples using a t-test. Categorical data were analyzed using the chi-square test. The correlation between ultrasound examination parameters and pathological features of papillary thyroid carcinoma was analyzed using Spearman’s analysis. A P-value<0.05 indicated a statistically significant difference.

## RESULTS

**Fig.1:**
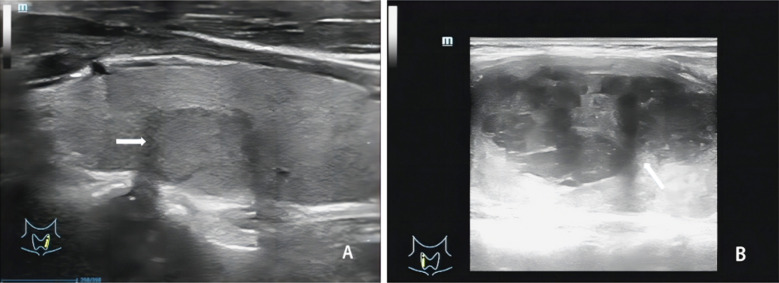
Ultrasound examination of benign and malignant ovarian tumors. A: The imaging features of benign group patients on ultrasound examination are mainly multiple masses with a diameter of<3cm and accompanied by high echogenicity; B: The imaging features of malignant group patients on ultrasound examination are mainly solitary masses with a diameter of ≥ 3cm and accompanied by hypoechogenicity.

**Fig.2:**
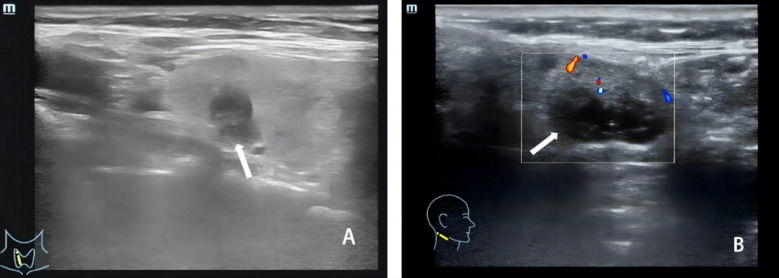
Ultrasound examination of lymph node metastasis in thyroid malignant tumors. A: The tumor has a small volume and no lymph node metastasis is observed in the neck; B: The tumor has a large volume, with abnormal lymph node metastasis visible in the neck. The normal lymph node structure disappears, and the boundary between the cortex and medulla is unclear. Calcification can be detected in the cortex of lymph nodes, and blood flow is disordered.

**Fig.3:**
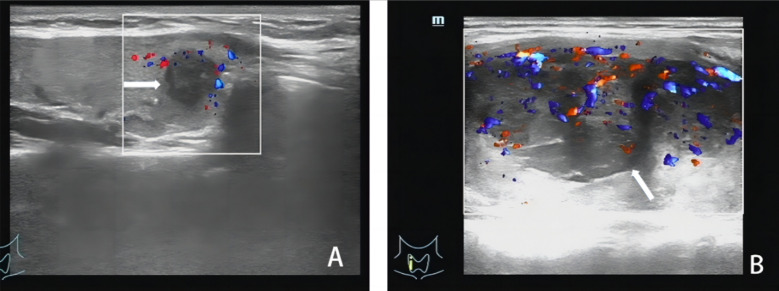
Ultrasound examination images of different stages of thyroid malignant tumors. A: Low velocity blood flow in stage II patients; B: High blood flow in stage IV patients.

The baseline characteristics of the study population are shown in Table-I. There were no significant differences in age, gender, tumor diameter, and body mass index (BMI) between the malignant (papillary thyroid carcinoma, PTC) and benign groups (*P > 0.05*). The ultrasound characteristics of PTC and benign thyroid lesions are presented in Fig.1. The peak systolic blood flow velocity (PSV), pulsation index (PI), and resistance index (RI) were significantly higher in the malignant group, while peak intensity (Peak), mean transit time (MTT), time to peak (TTP), and area under the curve (AUC) were significantly lower compared to the benign group (*P < 0.05*) (Table-II). In patients with lymph node metastasis (LNM) (Fig.2) and stage III-IV PTC (Fig.3), the PSV, PI, and RI were significantly higher, while Peak, MTT, TTP, and AUC were significantly lower compared to patients without LNM and those with stage I-II disease *(P < 0.05)*.

**Table-I:** Comparison of baseline data between two groups.

Group	Gender (male/female)	Age (year)	Lesion diameter (cm)	Body mass index (kg/m²)
Malignant group (n=89)	57/32	54.97±10.78	2.59±1.24	22.87±3.12
Benign group (n=89)	50/39	53.02±11.34	2.62±1.19	23.02±3.08
*χ^2^*/*t*	1.148	1.172	-0.167	-0.305
*P*	0.284	0.243	0.868	0.761

**Table-II:** Comparison of ultrasound examination parameters between malignant and benign groups.

Group	PSV (cm/s)	PI	RI	Peak (dB)	MTT (s)	TTP (s)	AUC
Malignant group (n=89)	39.53±6.40	0.90±0.14	2.23±0.51	12.47±1.45	0.88±0.13	0.92±0.15	739.54±77.34
Benign group (n=89)	24.13±3.63	0.62±0.11	1.42±0.36	20.51±4.56	1.06±0.17	1.21±0.20	997.30±134.05
*t*	19.746	14.836	12.241	15.851	7.935	10.943	15.713
*P*	<0.001	<0.001	<0.001	<0.001	<0.001	<0.001	<0.001

***Note:*** PSV: peak systolic blood flow velocity; PI: pulsation index; RI: resistance index; Peak: peak intensity; MTT: mean transit time; TTP: time to peak; AUC: area under curve. S: second.

**Fig.4:**
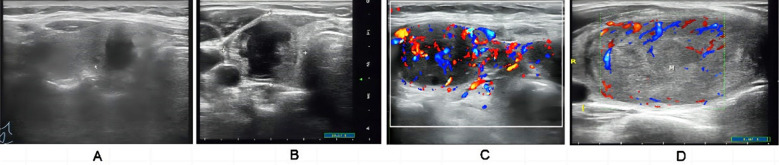
Ultrasound images of PTC patients with different types of gene mutations. A: BRAF mutations are characterized by vertical orientation, irregular edges, extremely low echogenicity, microcalcifications, and silent halos; B: RAS mutations are characterized by low echogenicity, oval shape, partially cystic changes, and no microcalcifications; C: RET/PIC rearrangement shows extremely low echogenicity, diffuse changes, membrane invasion with central lymph node metastasis; D: TERT mutation manifests as hypoechoic, margin of lobules, palpable invasion, and early central lymph node metastasis.

Genetic profiling of the excised PTC tissues revealed that 79 out of 89 patients had mutations. Table III shows the mutation breakdown: five patients with a TERT gene mutation, nine with RET PTC gene rearrangement, 15 with an RAS gene mutation, and 50 with a BRAF gene mutation. Significant differences were found in the PSV, PI, RI, Peak, MTT, TTP, and AUC among patients with different gene mutations (*P < 0.0001;* Table-III). The unique ultrasound manifestations in thyroid glands of patients with BRAF, TERT, RAS, and RET PTC mutations is illustrated in Fig-4.. Spearman’s correlation analysis showed that PSV, PI, and RI were significantly positively correlated with LNM, disease stage, and gene mutations in PTC patients. In contrast, Peak, MTT, TTP, and AUC were significantly negatively correlated with these factors (*P < 0.05*) (Table-IV).

**Table-III:** Comparison of ultrasound examination parameters among patients with different pathological characteristics in the malignant group.

Pathological characteristics		n	PSV (cm/s)	PI	RI	Peak (dB)	MTT (s)	TTP (s)	AUC
Lymph Node Metastasis	No	51	31.42±5.31	0.81±0.13	1.95±0.45	15.97±2.98	1.01±0.15	1.15±0.19	801.43±95.23
	Yes	38	44.42±7.06	0.99±0.18	2.42±0.55	10.06±1.21	0.81±0.12	0.87±0.13	711.35±62.45
	*t*		9.919	5.480	4.431	11.525	6.761	7.818	5.071
	*P*		<0.001	<0.001	<0.001	<0.001	<0.001	<0.001	<0.001
Disease Staging	I~II	42	30.95±5.62	0.79±0.15	1.98±0.52	16.02±3.11	0.99±0.17	1.13±0.21	799.71±98.13
	III~IV	47	43.78±7.21	0.98±0.19	2.45±0.61	9.97±1.15	0.80±0.11	0.84±0.14	706.24±64.37
	*t*		9.436	5.272	3.916	11.411	6.011	7.374	5.106
	*P*		<0.001	<0.001	<0.001	<0.001	<0.001	<0.001	<0.001
Gene mutation	TERT gene mutation	5	46.67±6.15	1.09±0.20	2.96±0.63	8.81±1.35	0.75±0.10	0.72±0.12	621.13±49.26
	RET PTC gene rearrangement	9	42.11±5.39	0.97±0.14	2.42±0.54	9.89±1.54	0.86±0.09	0.86±0.15	652.07±53.13
	RAS gene mutation	15	36.78±5.19	0.88±0.12	1.93±0.42	11.21±2.02	0.95±0.12	0.97±0.16	696.71±60.54
	BRAF gene mutation	50	32.05±4.64	0.77±0.10	1.81±0.37	13.51±3.12	1.03±0.14	1.09±0.17	758.60±87.02
	No mutation	10	29.84±5.51	0.71±0.06	1.79±0.42	16.38±3.54	1.11±0.16	1.24±0.23	821.56±90.33
	*F*		18.664	17.493	11.910	10.828	10.723	11.829	9.990
	*P*		<0.001	<0.001	<0.001	<0.001	<0.001	<0.001	<0.001

***Note:*** PSV: peak systolic blood flow velocity; PI: pulsation index; RI: resistance index; Peak: peak intensity; MTT: mean transit time; TTP: time to peak; AUC: area under curve. S: second.

**Table-IV:** Analysis of the correlation between ultrasound examination parameters and pathological characteristics of papillary thyroid carcinoma.

Pathological characteristics	PSV (cm/s)	PI	RI	Peak (dB)	MTT (s)	TTP (s)	AUC

	r/P	r/P	r/P	r/P	r/P	r/P	r/P
Disease Staging	0.530/<0.001	0.603/<0.001	0.551/<0.001	-0.473/<0.001	-0.427/<0.001	-0.506/<0.001	-0.403/<0.001
Lymph node metastasis	0.523/<0.001	0.544/<0.001	0.418/<0.001	-0.450/<0.001	-0.410/<0.001	-0.411/<0.001	-0.452/<0.001
Gene mutation	0.374/<0.001	0.496/<0.001	0.433/<0.001	-0.388/<0.001	-0.434/<0.001	-0.409/<0.001	-0.394/<0.001

***Note:*** PSV: peak systolic blood flow velocity; PI: pulsation index; RI: resistance index; Peak: peak intensity; MTT: mean transit time; TTP: time to peak; AUC: area under curve. S: second.

Among the 89 PTC patients, 27 had disease recurrence, and five died during the follow-up period. These 32 cases were categorized as the poor prognosis group, while the remaining 57 cases were classified as having a good prognosis. The PSV, PI, and RI were significantly higher in the poor prognosis group, while Peak, MTT, TTP, and AUC were significantly lower compared to the good prognosis group (*P < 0.05*) (Table-V).

**Table-V:** Comparison of ultrasound examination parameters in patients with different prognosis.

Prognosis	n	PSV (cm/s)	PI	RI	Peak (dB)	MTT (s)	TTP (s)	AUC
Poor prognosis	32	38.42±6.32	0.87±0.16	2.19±0.49	11.36±1.34	0.86±0.15	0.90±0.13	728.43±76.23
Good prognosis	57	23.09±3.44	0.59±0.10	1.32±0.25	16.37±4.33	1.04±0.16	1.19±0.18	986.21±123.14
*t*		14.847	10.162	11.105	6.362	5.207	8.008	10.729
*P*		<0.001	<0.001	<0.001	<0.001	<0.001	<0.001	<0.001

***Note:*** PSV: peak systolic blood flow velocity; PI: pulsation index; RI: resistance index; Peak: peak intensity; MTT: mean transit time; TTP: time to peak; AUC: area under curve. S: second

## DISCUSSION

The results of this study indicate that the PSV, PI, and RI in patients with papillary thyroid carcinoma were higher than those in patients with benign tumors. In contrast, Peak, MTT, TTP, and AUC were lower in the tumor group compared to the benign group. Additionally, we showed a correlation between the genetic profiles of papillary thyroid carcinoma patients and US parameters. Our findings clearly demonstrate that Ultrasound examination parameters can be used to differentiate thyroid tumors. Previous research by Li et al.^18^ showed that US blood flow examination effectively clarifies differences in blood flow status, internal echoes, boundaries, and morphology between benign and malignant thyroid tumors, providing an objective basis for differential diagnosis. Lei et al.^19^ confirmed significant differences in ultrasound examination parameters (blood flow characteristics) and ultrasound features (such as echo intensity, edge shape, lesion volume, etc.) between papillary thyroid carcinoma patients and benign tumors that can be effectively used for distinguishing between benign and malignant tumors and for pathological evaluation.^19^

Fang et al.^20^ further demonstrated significant differences in peak intensity, peak time, and centrifugal perfusion in papillary thyroid carcinoma patients compared to patients with benign thyroid nodules. Our results, in conjunction with these studies, support US examination as a valuable tool for distinguishing between benign and malignant thyroid tumors. Gao et al.^21^ examined the role of US examination in diagnosing and evaluating PTC, finding significant differences in color Doppler flow imaging (CDFI) blood flow grading between malignant and benign tumors. In their study, the RI and Vmax of PTC patients were significantly higher than those of patients with benign thyroid nodules, and the sensitivity, accuracy, and specificity of US examination for diagnosing PTC reached 81.93%, 87.40%, and 97.73%, respectively.

Wang et al.^22^ found correlations between pre-operative contrast-enhanced ultrasound (CEUS) parameters and biological characteristics of PTC, which were valuable in assessing prognosis after radiofrequency ablation. Additionally, Yi L et al.^23^ examined the role of ultrasound elastography in understanding the histologic characteristics of PTC, supporting the association between ultrasound features and tumor biology. A study by Jiang^24^ that explored the correlation between ultrasound parameters and pathological characteristics of PTC showed that the levels of PI, RI, PSV, and other indicators in papillary thyroid carcinoma patients are higher than those in patients compared to those with benign thyroid diseases.

These results and conclusions are consistent with our study to a certain extent and further confirm the high application value of ultrasound in diagnosing and evaluating thyroid malignant tumors and in the ability to effectively distinguish between benign and malignant properties of PTC.^18^–^20^ We hypothesize that, compared to benign thyroid tumors, PTC lesions have a small number of new blood vessels with relatively disordered distribution, lacking mature functions, which hinders nutritional, oxygen, and other requirements that are necessary for the high growth of tumor cells. The resulting ischemia and hypoxia, in turn, may lead to non-enhancement or low levels of lesion enhancement, manifested as shortened MMT and TTP, along with increased PI and RI.^18^–^20^,^24^ This theory is supported by the study by Cao et al.^25^ which, through pathological examination, demonstrated a relatively small number of microvessels in PTC lesions. We also conducted a comparative study of US examination parameters in patients with different pathological characteristics. Our results showed that PSV, PI, and RI in patients with LNM and stage III-IV PTC were higher, while Peak, MTT, TTP, and AUC were lower than those without LNM and stage I-II cancer. Additionally, there were significant differences in the levels of various indicators among patients with different gene mutations.

This close correlation indicates that US examination can also be used for clinical evaluation of pathological features of PTC, guiding physicians to develop targeted intervention plans, ensuring treatment effectiveness and safety, and avoiding overtreatment or improper treatment.^26^ The observed correlation between US parameters and pathological characteristics of the thyroid tumors may be due to a disordered arrangement of the new blood vessels in the PTC lesion. These vascular changes lead to variations in relevant parameters, with the amplitude of these changes increasing with disease stage and LNM. Our study further demonstrates that US examination parameters vary among patients with different prognoses, suggesting that US examination can be used for prognostic evaluation of PTC, offering valuable guidance for clinical intervention.^27^–^29^

In addition to the pathological characteristics of the disease, our study provides clear evidence of the link between US examination parameters and specific gene profiles of PTC. Current research identifies point mutations in *BRAF*, *RAS*, *TERT*, *and RET*/*PTC* genes as the leading genetic causes of PTC.^30^,^31^ A mutation in *BRAF* that encodes MAPK pathway cytoplasmic kinase was shown to downregulate the expression of thyroglobulin and thyroperoxidase genes^32^,^33^ and is associated with higher tumor aggressiveness and worse prognosis.^34^ Mutations in the RAS genes that encode proteins of the MAPK/PI3K/AKT pathway may predispose benign thyroid tumors progression to carcinoma.^35^,^36^ Chromosomal rearrangement *RET*/*PTC* is associated with more aggressive tumor behavior and frequent metastatic dissemination.^37^

Finally, mutations in the TERT gene that encodes for telomerase are found in patients with disseminated PTC and are associated with high tumor aggressiveness.^38^,^39^ In our study, genetic mutations in *BRAF*, *RAS*, *TERT*, and *RET*/*PTC* were associated with markedly elevated PSWV, RI and PI. At the same time, PEAK, MTT, TTP, and AUC in such patients were significantly lower compared to PTC patients without genetic alterations. Ultrasound images of PTC patients with different gene mutations revealed unique characteristics associated with each mutation.

### Limitations:

It is a single-center, retrospective analysis with a small sample size, which may limit the generalizability of the findings. Additionally, ultrasound parameters may be influenced by operator experience, machine settings, and patient factors, highlighting the need for standardized protocols. The follow-up period was also limited to six months, which restricts the ability to assess the long-term prognostic value of ultrasound. To validate these findings and further enhance clinical applicability, larger, multicenter studies with longer follow-up and standardized imaging protocols are recommended.

## CONCLUSION

This study demonstrates that ultrasound examination parameters are significantly associated with the pathological characteristics and prognosis of PTC. Specifically, PSV, PI, and RI positively correlated with lymph node metastasis and advanced disease stage, while Peak, MTT, TTP, and AUC showed negative correlations. These ultrasound parameters not only aid in distinguishing malignant from benign thyroid nodules but also offer valuable insights into disease staging and prognosis. Thus, ultrasound examination proves to be a highly valuable, non-invasive tool for diagnosing, evaluating, and prognosticating PTC. Future studies, particularly multicenter and prospective trials, are needed to confirm these findings and further standardize ultrasound imaging techniques for broader clinical application in the management of PTC.

### Authors’ contributions:

**LY:** Conceived and designed the study. Literature search

**LY, YL** and **ZL:** Collected the data, performed the analysis and critical review.

**LY:** Critical analysis, Was involved in the writing of the manuscript.

All authors have read, approved the final manuscript and are responsible for the integrity of the study.
